# Epidemiology and incidence of oral squamous cell carcinoma in the Iraqi population over 5 years (2014–2018)

**DOI:** 10.1002/hsr2.1205

**Published:** 2023-04-11

**Authors:** Muhanad L. Alshami, Mohammed Abbood Al‐Maliky, Ali Arkan Alsagban, Ali J. Alshaeli

**Affiliations:** ^1^ Department of Dentistry Dijlah University College Baghdad Iraq; ^2^ Department of Dentistry Al‐Hadi University College Baghdad Iraq; ^3^ College of Dentistry University of Baghdad Baghdad Iraq

**Keywords:** epidemiology, incidence, oral squamous cell carcinoma

## Abstract

**Background:**

Oral squamous cell carcinoma is one of the most common and life‐threatening neoplasms worldwide, and is responsible for approximately 90% of all oral malignancies.

**Aim:**

This study was aimed at providing updated information on oral squamous cell carcinoma in all Iraqi governorates for the 5‐year period from 2014 to 2018, including the annual incidence and demographic variables.

**Materials and Methods:**

The total number of oral squamous cell carcinoma cases in Iraq, along with associated demographic information (age, sex, and site), for the 5‐year period from 2014 to 2018 was obtained. The statistical analysis consisted of descriptive analysis, including frequency, percentage, and mean ± standard deviation. A *χ* ^2^ test was performed to compare frequencies between male and female patients, among age groups, and among different OSCC sites. The *χ* ^2^ test was also used to assess the association of each OSCC site with age and sex. The significance threshold was set at *p* < 0.05, and the confidence interval was set at 95%. The incidence rate of oral squamous cell carcinoma for each year was calculated by dividing the number of OSCC cases per year by the population of Iraq, then multiplying the result by 100,000.

**Results:**

A total of 722 cases were recorded. Statistically, oral squamous cell carcinoma was found to be more prevalent in males and individuals over 40 years of age. The tongue was the most common site of occurrence. Lip squamous cell carcinoma cases were high in males. The incidence rate of oral squamous cell carcinoma was estimated to be 0.4 per 100,000 people.

**Conclusion:**

Males and older people are at relatively higher risk of developing oral cancer. The tongue is the most affected site, but any site in the oral cavity may be involved. Further exploration of the causes of oral malignancy in Iraq is necessary to improve prevention strategies.

## 1 INTRODUCTION

Oral squamous cell carcinoma (OSCC), a malignant neoplasm of the oral cavity, is associated with high morbidity and mortality.^1^ OSCC is one of the most frequent malignancies worldwide, and it accounts for 90% of all oral cavity cancers.^2^ Multiple risk factors have been documented to be associated with OSCC, including smoking, alcohol intake, infection, sun exposure, poor oral hygiene, chronic irritability, and genetic disorders.^3^, ^4^ Many studies have indicated that males are more commonly affected by OSCC than females, and older people are believed to be at the highest risk of developing OSCC.^5^, ^6^ OSCC may affect the mucosa of all anatomical sites of the oral cavity.^7^ Clinically, OSCC usually manifests as ulcers, exophytic tumors, or patches of leukioplakia or erythoplakia.^8^ The prognosis of OSCC is affected by the stage and location of the cancer, and the overall health of the patient. The 5‐year survival rate for people with early‐stage OSCC is approximately 80%–90%, but is only approximately 30%–50% for people with advanced‐stage OSCC.^9^, ^10^ Epidemiological studies are important for understanding the prevalence, demographic characteristics, and incidence of cancer, as well as the risk factors for specific types of cancer. In addition, epidemiological studies enable evaluation of the effectiveness of cancer control strategies. Incidence refers to the number of new cases of cancer in a specific period. The outcomes of epidemiological studies can be used to guide the development and implementation of cancer prevention and control programs.^11^, ^12^ The last nation‐wide epidemiological study on OSCC in Iraq was for the period from 2001 to 2013, but that study did not cover all of Iraq, because the Kurdistan region was not included.^13^ Therefore, the objectives of the present study were to provide updated information on OSCC in all Iraqi governorates for 5 years (2014–2018), including the annual incidence and demographic variables (age, sex, and site).

## 2 MATERIALS AND METHODS

The current study was a retrospective cohort study conducted in Iraq between September 1, 2021 and April 1, 2022. Before initiation of the study, ethical approval (ref: 1926–8/26/2021) was obtained from the ethical committee of Dijhla University College. The study design was based on those of previous studies.^14^, ^15^

The number of all OSCC cases with confirmed diagnosis in all Iraqi governorates, and related information (age, sex, and site of lesion), over the course of 5 years (2014–2018) were obtained from the archive of the Iraqi Cancer Board (ICB), Ministry of Health, Iraq. ICB consent as obtained to use of the data for academic purposes.

The ICB relied on both governmental and private histopathological laboratories in each Iraqi governorate to collect annual data on OSCC cases.

For estimating the incidence of OSCC, the annual population census of Iraq from 2014 to 2018 was obtained from the official website of the Central Statistical Organization of the Iraqi Ministry of Planning.^16^

The sexes were classified as male and female, and the age was calculated by year and divided into groups >40 and ≤40 years of age.

The locations of OSCC cases were classified as buccal mucosa, floor of the mouth, gingiva (gingiva and retromolar pad), lips (upper and lower lips), unspecified sites, palate (hard and soft palate), and tongue.

## 3 STATISTICAL ANALYSIS

Statistical analysis was conducted in SPSS software (version 14) and included descriptive analysis and inferential analysis. The descriptive analysis included the frequency, percentage, and mean ± standard deviation (SD). The inferential analysis first included *χ* ^2^ tests to compare frequencies between males and females, among age groups, and among the sites of OSCC. Subsequently, the association of each site of OSCC with age and sex was tested. Significance was established at *p* < 0.05, and the confidence intervals were set to 95%.

The incidence of OSCC per 100,000 people for each year was calculated as the number of OSCC cases divided by the number of Iraqis at risk of OSCC, multiplied by 100,000.^17^

## 4 RESULTS

**Figure 1:**
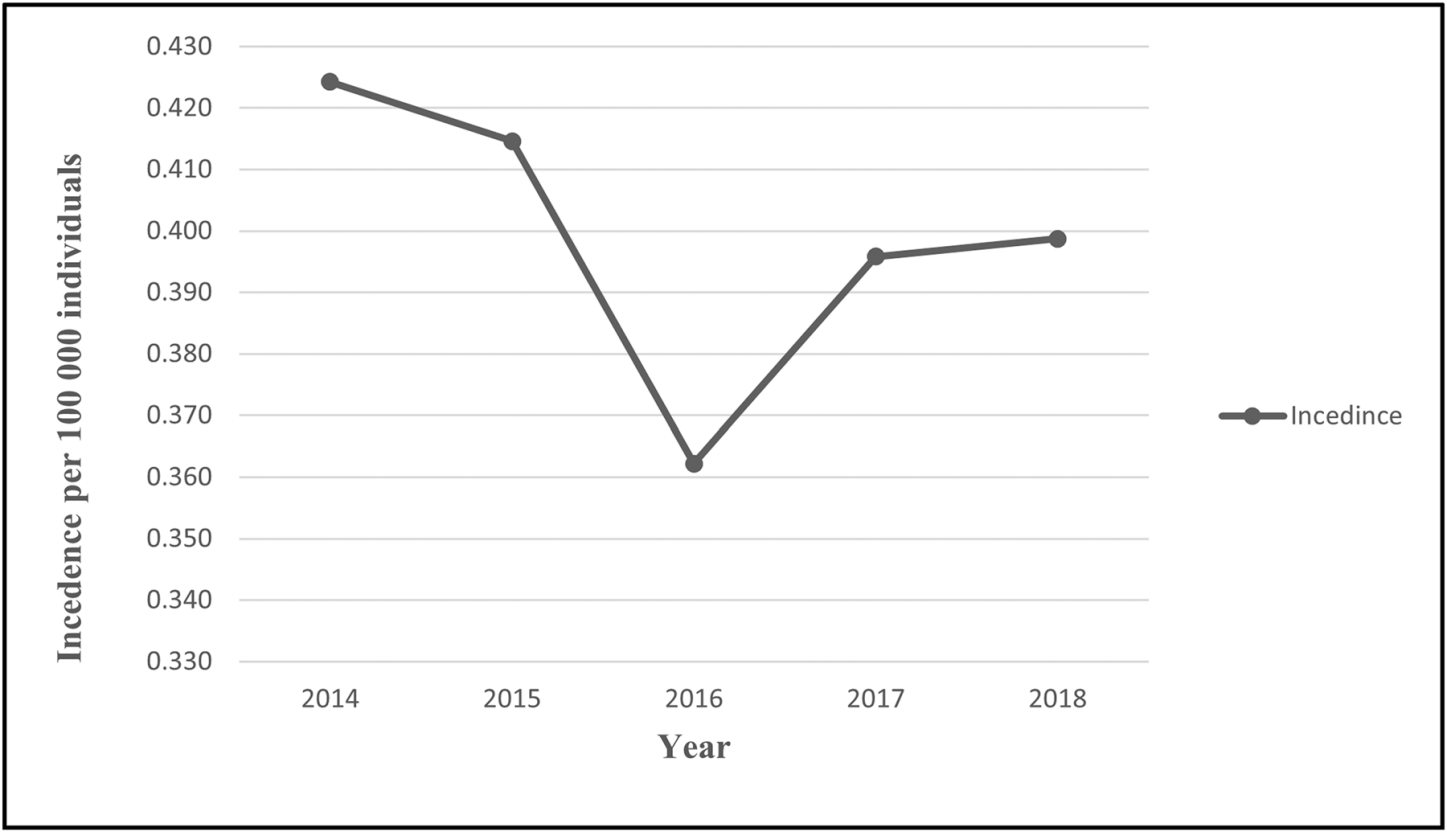
The annual incidence of oral squamous cell carcinoma per 100,000 people in Iraq from 2014 to 2018. The incidence was calculated by dividing the number of cases by the population at risk, then multiplying the result by 100,000.

The total number of OSCC cases in Iraq during the 5‐year period (2014–2018) was 722. The number of males (389) was significantly higher than that of females (333), and the male‐to‐female ratio was 1.2:1. The prevalence of OSCC was significantly higher among individuals older than 40 years (*p* ˂ 0.00). The most common sites for OSCC were the tongue, followed by the lips. Gingiva was the least frequent site affected by OSCC. Table 1 describes the distribution of sexes, age groups, and cancer sites. Table 2 shows the number of OSCC cases per year. The incidence of OSCC in each year (2014–2018) ranged from 0.362 to 0.424 per 100,000 individuals, and the average incidence was 0.399 (Figure 1).

**Table 1:** Oral squamous cell carcinoma distribution in Iraq from 2014 to 2018, according to site and demographic variables (sex and age).

Variables	Frequency, %	CI^b^	*p* Value*
Sex			
Male	389, 53.9%	0.021–0.048	**0.04**
Female	333, 46.1%		
M:F^a^	1.2:1		
Age (years)			
≤40	82, 11.4%	0.000–0.004	**˂0.001**
>40	640, 88.6%		
Mean age ± SD	58.09 ± 15.51		
Minimum–maximum	4–99		
Site			
Buccal mucosa	41, 5.7%	0.000–0.004	**˂0.001**
Floor of the mouth	31, 4.3%		
Gingiva	22, 3.0%		
Lip	131, 18.1%		
Nonspecified site	100, 13.9%		
Palate	35, 4.8%		
Tongue	362, 4.8%		

^a^ Male to female ratio.

^b^ Confidence intervals (CI) at 95%.

* Bold format indicates significance at *p* ˂ 0.05 by *χ* ^2^ test.

**Table 2:** Number of oral squamous cell carcinoma cases per year in Iraq from 2014 to 2018.

Year	Population^a^	OSCC cases^a^
2014	34,411,949	146
2015	35,212,600	146
2016	36,169,123	131
2017	37,139,519	147
2018	38,124,182	152

^a^ Frequency.

No associations between male or female sex and cancer site were observed, except for the lips. The frequency of OSCC on the lips was significantly higher in males than in females. The prevalence of OSCC in all anatomical sites was high in patients >40 years of age. Table 3 shows the association of cancer site with demographic variables (sex and age).

**Table 3:** Associations of oral squamous cell carcinoma site with age and sex.

Site	Sex				Age (year)			
	Male^a^	Female^a^	CI^b^	*p* Value*	≤40^a^	>40^a^	CI^b^	*p* Value*
Buccal mucosa	20, 48.8%	21, 51.2%	0.930–1.000	0.59	3, 7.3%	38, 92.7%	0.000–0.070	**˂0.001**
Floor of the mouth	20, 64.5%	11, 35.5%	0.054–0.333	0.11	3, 9.7%	28, 90.3%	0.000–0.092	**˂0.001**
Gingiva	9, 40.9%	13, 59.1%	0.385–0.796	0.39	5, 22.7%	17, 77.3%	0.021–0.343	**0.01**
Lip	81, 61.8%	50, 38.2%	0.000–0.023	**0.007**	15, 11.5%	116, 88.5%	0.000–0.023	**˂0.001**
Nonspecified site	57, 57.0%	43, 43.0%	0.113–0.267	0.16	5, 5.0%	95, 95.0%	0.000–0.030	**˂0.001**
Palate	16, 45.7%	19, 54.3%	0.704–0.953	0.61	6, 17.1%	29, 82.9%	0.000–0.082	**˂0.001**
Tongue	186, 51.4%	176, 48.6%	0.620–0.717	0.59	45, 12.4%	317, 87.6%	0.000–0.008	**˂0.001**

^a^ Male to female ratio.

^b^ Confidence intervals (CI) at 95%.

* Bold format indicates significance at *p* ˂ 0.05 by *χ* ^2^ test.

## 5 DISCUSSION

The results of the current study indicated a higher incidence of OSCC among males than females. This finding is in agreement with those from prior studies.^18^, ^19^, ^20^ A plausible explanation for this result may be that the documented prevalence of smoking and alcohol consumption in Iraq and other countries is higher among men than women.^21^, ^22^, ^23^, ^24^ Furthermore, the tendency for men to have occupations to located outdoors increases their exposure to carcinogenic elements, such as ultraviolet radiation and human papilloma virus.^25^, ^26^ The present investigation indicated that the male‐to‐female ratio was 1.2:1, a ratio similar to the 1.4:1 for OSCC in Iraq from 2001 to 2013 reported by Enas and colleagues.^27^ Previous epidemiological studies conducted in other countries, such as India,^28^ Brazil,^29^ Taiwan,^30^ México,^31^ Qatar,^15^ and Jordan,^32^ have also reported a high prevalence of OSCC in males.

Most cases of OSCC were identified in individuals older than 40 years, and the average patient age was in the fifth decade, in concordance with the results of prior investigations conducted in Iraq,^33^ Pakistan,^34^ Mexico,^31^ Iran,^35^ and Brazil.^18^ Aging is considered to contribute to the development of OSCC, owing to multiple interrelated factors. As individuals advance in years, they become more susceptible to the risk factors associated with OSCC, such as exposure to tobacco and alcohol use.^36^ Furthermore, the decline in the body's natural defense mechanisms with age increases the vulnerability of older individuals to cancer development.^37^ Moreover, the ability of the body to repair DNA damage decreases with age, thus potentially further contributing to the onset of OSCC.^38^ In addition, older individuals tend to have insufficient awareness of oral health, which is a risk factor for oral cancer.^39^ Consequently, the incidence of OSCC increases with age, thus making age a critical factor in OSCC development.

The tongue was found to be the most common site of OSCC in the present study, in agreement with previous research conducted in Iraq and other parts of the world.^29^, ^40^ The high incidence rate of tongue cancer is explained by the tongue's being subjected to repeated friction and trauma caused by sharp or misaligned teeth or improperly designed dental prostheses. Additionally, the tongue is located near areas that are often exposed to carcinogens, such as tobacco and alcohol, thereby increasing the risk of OSCC.^41^ In contrast, a previous study in Taiwan has indicated that the buccal mucosa is a common site of squamous cell carcinoma. This finding has been attributed to the habit of betel quid chewing.^42^

The present study determined that the average incidence of OSCC over the 5‐year period of 2014–2018 was 0.4 per 100,000 people. This result is consistent with the findings of Shahrour and colleagues, who have reported an incidence of OSCC in Syria of 0.5 per 100,000 people.^43^ However, the observed incidence of OSCC in this study is lower than those reported in China,^44^ Japan,^45^ and Korea ^46^ (2.9, 3.8, and 4.6 cases per 100,000 people, respectively). To our knowledge, this study is the first to comprehensively evaluate the incidence of OSCC across all governorates in Iraq.

The limitations of the current study include the lack of direct examination and sufficient information regarding tumor stage and grade, as well as prognosis. Additional analogous studies are recommended to facilitate comparison of outcomes.

## 6 CONCLUSION

Males and older individuals are at risk of OSCC. The most common site for OSCC is the tongue. To decrease the incidence of OSCC, efforts must be made to increase community awareness regarding OSCC risk factors. Periodic oral examinations and screenings for oral cancer, as well as education on self‐examination of the oral cavity, are essential for early detection of OSCC.

## AUTHOR CONTRIBUTIONS

**Muhanad L. Alshami**: Data curation; formal analysis; software; visualization; writing—review & editing. **Ali Arkan Alsagban**: Investigation; validation. **Ali J. Alshaeli**: Conceptualization; methodology; visualization; writing—original draft.

## CONFLICT OF INTEREST STATEMENT

The authors declare no conflict of interest.

## ETHICS STATEMENT

Ethical approval for this study was obtained in accordance with the Declaration of Helsinki.

## TRANSPARENCY STATEMENT

The lead author Muhanad L. Alshami affirms that this manuscript is an honest, accurate, and transparent account of the study being reported; that no important aspects of the study have been omitted; and that any discrepancies from the study as planned (and, if relevant, registered) have been explained.

## Data Availability

The data that support the findings of this study are available from the corresponding author upon reasonable request.
